# Bullying among medical residents in a large public hospital: a cross-sectional study

**DOI:** 10.1590/1806-9282.20251808

**Published:** 2026-06-26

**Authors:** Luiz Antônio de Oliveira, Aleida Nazareth Soares, Renata Rocha Barreto Giaxa, Rosa Malena Delbone, Alexandre Sampaio Moura

**Affiliations:** 1 Faculdade Santa Casa de Belo Horizonte – Belo Horizonte (MG), Brazil.; 2 Faculdade de Ciências Médicas da Santa Casa de São Paulo – São Paulo (SP), Brazil.

**Keywords:** Bullying, Risk factors, Medical education, Medical residency, Occupational health

## Abstract

**OBJECTIVE:**

Bullying is a prevalent issue in medical residency worldwide, yet data from Latin America remain limited. The aim of this study was to assess the prevalence of bullying and its associated factors among medical residents in a public hospital in Brazil.

**METHODS:**

A cross-sectional study was conducted with 82 medical residents. Bullying was assessed using the Negative Acts Questionnaire-Revised.

**RESULTS:**

There was a prevalence of bullying ranging from 45.1% (Leymann’s criteria) to 51.2% (Einarsen, Hoel, and Notelaers criteria). The overall median Negative Acts Questionnaire-Revised score was 33 (interquartile range: 27–42), with no significant difference between first-year residents and later-year residents (28.0 vs. 34.5; p=0.06). However, first-year residents reported lower median scores for some bullying domains, including “Work-Related Bullying” (p=0.04), “Personal and Professional Disqualification” (p=0.03), and “Physical Intimidation” (p=0.02). Only 7% of residents self-reported feeling bullied. No associations were found between bullying and sex or specialty.

**CONCLUSION:**

These findings indicate a high prevalence of bullying in the medical residency programs studied, particularly among more advanced trainees. The large gap between assessed and self-perceived bullying suggests a lack of awareness about what constitutes harassment.

## INTRODUCTION

Bullying refers to repeated and long-lasting behaviors that may cause psychological harm, often manifested through verbal aggression, exclusion from team activities, obstruction of tasks, or disqualification of professional identity^1^.

The consequences of bullying can extend beyond personal suffering, leading to financial strain, reduced job performance, organizational dysfunction, and diminished productivity^1^.

Bullying in healthcare presents specific characteristics, such as frequent underreporting and the perception of its occurrence as a natural part of training, which perpetuate a cycle of aggression and silence^2^. International studies indicate that bullying is highly prevalent in residency training, varying from 37 to 73%, and women and more advanced trainees tend to be more frequently exposed^2,3,4,5^.

In Brazil, there are few studies on the prevalence and factors associated with bullying during medical training. A study conducted at a university hospital in São Paulo found that approximately 90% of medical residents experienced some form of bullying during their first year of training, with negative impacts on the educational environment and their mental health. The most common types of bullying identified in this study were social isolation and humiliation^6^.

We sought to estimate the prevalence of bullying among medical residents at a large public hospital in Brazil and analyze the factors contributing to its occurrence.

## METHODS

### Study design and setting

This observational cross-sectional study was conducted between March and May 2024, at Santa Casa BH (SCBH). It was approved by the Research Ethics Committee (CAAE: 76491423.6.0000.5138) and conducted in accordance with the Declaration of Helsinki.

### Participants and recruitment

The study included a convenience sample of medical residents enrolled in various residency programs at SCBH. Residents who were on vacation, on leave, or completing an external rotation at the time of data collection were excluded.

The sample size was calculated using GPower software (version 3.1.9.7). Considering a bullying prevalence of 63% among residents^2^, with a 10% margin of error, the required number of participants was estimated at 76.

All 497 medical residents from the 35 residency programs of the institution were eligible to participate, of whom 82 (16.5%) were included. All participants signed informed consent.

### Instruments and procedures

Data were collected using an online questionnaire. Bullying was assessed using the Brazilian version of the Negative Acts Questionnaire-Revised (NAQ-R), which comprises 22 items^7^. To assess bullying, three criteria were applied. The first was Leymann’s criterion, which defines bullying as exposure to negative behaviors at least once a week for 6 months or more^8^. This definition emphasizes persistence and repetition as central elements for classifying workplace bullying.

The second criterion, proposed by Einarsen, Hoel, and Notelaers, distinguishes between types and intensities of negative behaviors and classifies individuals as “not bullied,” “occasionally exposed to bullying,” or “victims of bullying”^9^.

The third criterion was based on the self-labeling method proposed by Mikkelsen et al. and is based on a dichotomic response (yes/no) to an item that assesses the respondent’s self-perception of being a victim of bullying^10^.

To analyze the bullying pattern, we opted to use a validated structure with four factors: (1) work-related bullying; (2) personal bullying; (3) personal and professional disqualification; and (4) physical intimidation^9,11^. The prevalence of bullying for each of these factors was estimated using Leymann’s criterion, which involves exposure to at least one item of each factor at least once a week for 6 months or more^8^.

### Statistical analysis

Descriptive analyses were presented as absolute frequencies and percentages for categorical variables and as medians with interquartile ranges (IQRs) for continuous variables. Group comparisons for continuous measures were conducted with the nonparametric Mann-Whitney U test, while associations between categorical variables were examined using Fisher’s exact test. Relationships among bullying-related factors were explored through Spearman’s rank-order correlation. Statistical significance was defined as p<0.05. Analyses were conducted using SPSS software, version 25.0 (IBM Corp., Armonk, NY, USA).

## RESULTS

A total of 82 medical residents participated in the study. There was a female predominance among participants (67.1%), with a mean age of 29.6 years (standard deviation [SD]=4.4). Most participants were enrolled in the General Surgery program (25.6%), followed by Pediatrics (15.8%), Internal Medicine (6.1%), and Anesthesiology (4.8%). A total of 41 residents (50.0%) were enrolled in clinical specialties, 32 (39.0%) in surgical specialties, and 8 (9.7%) in mixed specialties. Regarding year of training, 22 (26.8%) were in the first year (R1), 22 (26.8%) in the second year (R2), and 38 (46.3%) were senior residents (R3 or R4).

**Figure 1:**
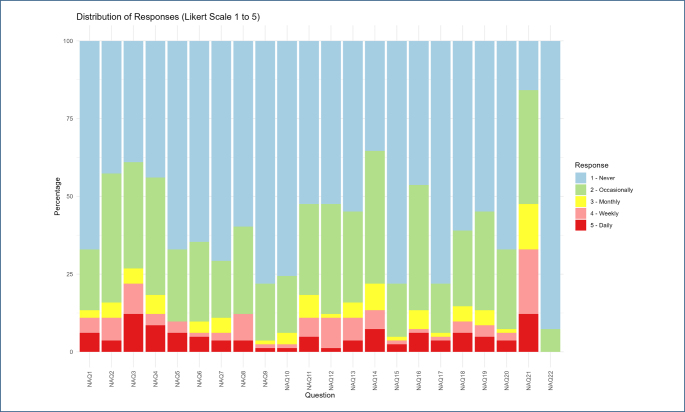
Distribution of responses for each item of the Negative Acts Questionnaire-Revised. Responses are presented as percentages on a 5-point Likert scale.

The distribution of responses in each item of the NAQ-R is shown in Figure 1. The overall prevalence of bullying in the sample varied depending on the criterion used. A total of 37 residents (45.1%) met Leymann’s criteria for bullying, and 42 (51.2%) met the Einarsen, Hoel, and Notelaers criteria (Table 1). Using the self-labeling criterion, only six participants (7.3%) considered themselves victims of bullying; of these, two (2.44%) reported that the bullying occurred “several times per week,” and four (4.88%) reported that it occurred daily.

**Table 1:** Prevalence of bullying among medical residents according to the criteria proposed by Leymann and Einarsen et al^8,9^.

Criteria	Category	n (%)
Leymann^8^	Overall bullying	37 (45.1%)
	Work-related bullying	31 (37.8%)
	Personal bullying	15 (18.3%)
	Personal and professional disqualification	24 (29.3%)
	Physical intimidation	5 (6.1%)
Einarsen et al.^9^	Not bullied	40 (48.8%)
	Occasionally bullied	25 (30.5%)
	Victims of bullying	17 (20.7%)

When stratifying the analysis by bullying factor, work-related bullying was the most prevalent (37.8%), followed by personal and professional disqualification (29.3%). A strong positive correlation was observed between these two factors (see Supplementary Table 1).

No significant differences were found in the prevalence of bullying (as defined by Leymann) between men and women (48.1 vs. 43.6%; p=0.700). No association was found between the prevalence of bullying and the year of training (p>0.999) or the specialty type (p=0.896).

The overall median bullying scores for R1s did not differ significantly from those observed for more advanced residents (28.0 [IQR: 24.0–36.0] vs. 34.5 [IQR: 28.0–43.0]; p=0.058). In the analysis stratified by bullying factor, the median “personal bullying” scores for R1s were lower than those of more experienced residents (8.0 [IQR: 7.0–12.0] vs. 10.0 [IQR: 8.0–13.0]; p=0.040). The median scores were also lower for R1s in the “personal and professional disqualification” and “physical intimidation” factors. For work-related bullying, no difference in median scores between R1 and more advanced residents was observed (12.5 [9.0–14.0] vs. 13.0 [11.0–16.0]; p=0.196) (Table 2).

**Table 2:** Bullying scores stratified by factor and residency training year.

Factor	Residency training year	Median	25th percentile	75th percentile	p-value
Overall bullying	R1	28.0	24.0	36.0	0.058
	R2, R3+R4	34.5	28.0	43.0	
Work-related bullying	R1	12.5	9.0	14.0	0.196
	R2, R3+R4	13.0	11.0	16.0	
Personal bullying	R1	8.0	7.0	12.0	**0.040**
	R2, R3+R4	10.0	8.0	13.0	
Personal and professional disqualification	R1	5.5	4.0	8.0	**0.028**
	R2, R3+R4	7.0	5.0	10.0	
Physical intimidation	R1	3.0	3.0	3.0	**0.021**
	R2, R3+R4	4.0	3.0	4.0	

Note: Values are medians and interquartile ranges (25th–75th percentile). The Mann-Whitney U test was used for comparisons. Bold p-values indicate statistical significance (p<0.05).

No statistically significant differences in overall bullying scores were found by sex or by specialty type (clinical vs. surgical).

## DISCUSSION

The overall prevalence of bullying found in our study was high, but consistent with the estimate of 64.1% reported in a meta-analysis on bullying in medical residency worldwide^12^.

Our findings were also consistent with prevalence estimates from other studies conducted in Brazil. A study involving medical and multiprofessional residents at a teaching hospital found a prevalence of bullying among physicians of 46.8%^13^. Similarly, a study by the Brazilian College of Surgeons reported a perceived prevalence of bullying of 49.1% during surgical training^14^. Another Brazilian study evaluating general surgery residents found that more than 50% of residents reported experiencing some form of bullying^15^.

These findings underscore the necessity for practical measures to mitigate bullying in academic settings, including comprehensive training for the multiprofessional team, the implementation of multiple reporting channels, and fostering an organizational culture of zero tolerance for bullying^16^. Residency program supervisors can help reduce bullying by fostering continuous professional development of clinical staff and establishing clear institutional policies against bullying^17^.

One interesting finding of this study was the correlation between different factors of bullying, including work-related bullying, personal and professional disqualification, and physical intimidation. This suggests that bullying behaviors may co-occur, reinforcing the need for an integrated approach to address this issue^1^.

Another relevant finding of this study was the marked discrepancy between bullying prevalence estimated using behavior-based criteria and the low proportion of residents who self-labeled as victims of bullying. Such low recognition may be influenced by institutional culture, in which hostile behaviors are normalized or perceived as inherent to medical training, as well as by fear of retaliation in hierarchical environments^18^. Additionally, this discrepancy may reflect a lack of insight into the abusive nature of certain interactions, particularly when bullying occurs in subtle or indirect forms.

Recent literature in psychology has increasingly focused on the concept of microaggressions^19^. In medical training, microaggressions can reinforce power imbalances and contribute to a culture where certain abusive behaviors are minimized, dismissed, or perceived as part of the learning process. Previous studies have emphasized how microaggressions can erode psychological safety and increase emotional distress among residents, particularly when combined with institutional silence or fear of retaliation^20^. Educational interventions aimed at improving recognition and awareness of workplace harassment have been proposed as strategies to address this gap^21^. Preventing bullying in medical residency may benefit the mental health of medical trainees and could contribute to patient safety and the quality of care^22^.

Our results show a similar distribution of bullying occurrences between male and female residents. This finding is consistent with a Brazilian study that also found no significant association between bullying and sex in residency programs^13^. This may reflect a broader trend within medical training environments rather than being necessarily attributed to specific features of organizational culture. Notably, women have increasingly asserted their roles in society and may be less intimidated by traditionally male-dominated environments than they were in the past^23^.

We found significant differences between novice and more experienced residents in personal bullying, personal and professional disqualification, and physical intimidation, with first-year residents reporting lower bullying scores. This finding may reflect either reduced exposure to bullying behaviors or lower recognition and reporting of such behaviors among R1s. In addition, they are often perceived as less threatening and more compliant, potentially resulting in fewer confrontational interactions with supervisors or senior colleagues. Limited experience may also contribute to difficulties in recognizing and labeling certain behaviors as bullying, and R1s may interpret hostile or demeaning interactions as inherent to the learning process. In contrast, more senior residents, who assume greater clinical responsibility and leadership roles, may be more frequently subjected to criticism, intimidation, or professional disqualification. These findings are consistent with those of a meta-analysis that identified a positive association between residency seniority and the prevalence of bullying^12^.

In our study, we did not find an association between medical specialty and bullying, in line with the findings from a previous study^2^. The authors emphasize that although some surgical specialties are traditionally perceived as having a more hostile environment, the data do not show differences across medical specialties.

A potential limitation of this study is the measurement of bullying. However, the NAQ-R questionnaire has been widely used in research on this topic in medical education. Additionally, the sensitive nature of the topic may have influenced the reported results. A systematic review revealed that residents underreport incidents of workplace or sexual harassment due to fear of retaliation^24^. To minimize the impact of this type of limitation, all data collection and responses were anonymous. Finally, because we used convenience sampling, selection bias might have distorted estimates of bullying prevalence, and the study estimates should be interpreted cautiously. However, Fevre et al. argue that while some people who feel more distressed about workplace relations might be more likely to respond to self-administered questionnaires on the topic, this bias would be offset by a countervailing general tendency to underreport negative workplace behaviors^25^.

## CONCLUSION

The prevalence of bullying among medical residents was high, mainly because of work-related bullying. First-year residents reported lower levels of bullying across several domains than more advanced trainees. No association was found between bullying and other factors such as sex, age, or specialty type. There was a large gap between assessed and self-perceived bullying, suggesting a lack of awareness about what constitutes harassment.

These findings underscore the responsibility of training institutions to address bullying in medical residency programs actively. Leadership engagement, the implementation of confidential and accessible reporting mechanisms, and the adoption of clear zero-tolerance policies might promote safe learning environments and ensure early recognition and effective management of bullying behaviors.

## Data Availability

The datasets generated and/or analyzed during the current study are available from the corresponding author upon reasonable request.
