# Supplementary material for: Bullying among medical residents in a large public hospital: a cross-sectional study
Source: Rev Assoc Med Bras (1992). 2026 Jun 26;72(3):e20251808. doi: 10.1590/1806-9282.20251808 (PMC13316863; doi:10.1590/1806-9282.20251808)
Supplement: Supplementary Table 1 [file 1806-9282-ramb-72-3-e20251808-suppl1.docx]

**Supplementary Table 1.** Spearman correlation coefficients (ρ) between the four harassment factors identified in the Negative Acts Questionnaire-Revised among medical residents (n=82).

| **Factors** | **Work-related harassment** | **Personal harassment** | **Personal and professional disqualification** | **Physical intimidation** |
| --- | --- | --- | --- | --- |
| Work-related harassment | – | 0.832* | 0.845* | 0.713* |
| Personal harassment | – | – | 0.761* | 0.662* |
| Personal and professional disqualification | – | – | – | 0.693* |
| Physical intimidation | – | – | – | – |

Note: All values represent Spearman’s ρ (rho). *p-value<0.001.
